# No association of the polymorphisms of the frizzled-related protein gene with peak bone mineral density in Chinese nuclear families

**DOI:** 10.1186/1471-2350-11-1

**Published:** 2010-01-01

**Authors:** Gao Gao, Zhen-Lin Zhang, Jin-Wei He, Hao Zhang, Hua Yue, Wei-Wei Hu, Jie-Mei Gu, Wen-Zhen Fu, Yun-Qiu Hu, Miao Li, Yu-Juan Liu, Jin-Bo Yu

**Affiliations:** 1 The Department of Osteoporosis, Metableic Bone Disease and Genetics Research Unit, Shanghai Jiao Tong University Affiliated Sixth People's Hospital. 600 Yi-Shan Rd, Shanghai 200233, PR China

## Abstract

**Background:**

The Wnt/beta-catenin signaling pathway plays an important role in skeletal development. Polymorphisms of frizzled-related protein (FRZB), an antagonist of this pathway, may generate variations in bone mineral density (BMD). In this study, we analyzed the association between *FRZB *genotypes and peak BMD variation in the spines and hips of two relatively large samples of Chinese female-offspring and male-offspring nuclear families.

**Methods:**

We recruited 1,260 subjects from 401 female-offspring nuclear families and 1,296 subjects from 427 male-offspring nuclear families and genotyped four tagging single nucleotide polymorphisms (tagSNPs) (rs6433993, rs409238, rs288324, and rs4666865) spanning the entire *FRZB *gene. The SNPs rs288326 and rs7775, which are associated with hip osteoarthritis, were not selected in this study because of their low minor allele frequencies (MAFs) in Chinese people. The quantitative transmission disequilibrium test (QTDT) was used to analyze the association between each SNP and haplotype with peak BMD in female- and male-offspring nuclear families.

**Results:**

In the female-offspring nuclear families, we found no evidence of an association between either single SNPs or haplotypes and peak BMD in the spine or hip. In the male-offspring nuclear families, no within-family association was observed for either SNPs or haplotypes, although a significant total association was found between rs4666865 and spine BMD (*P *= 0.0299).

**Conclusion:**

Our results suggest that natural variation in *FRZB *is not a major contributor to the observed variability in peak BMD in either Chinese females or males. Because ethnic differences in the *FRZB *genotypes may exist, other studies in different population are required to confirm such results.

## Background

Osteoporosis is a skeletal disorder characterized by fragile bones susceptible to low trauma fractures. One of the most important determinants for osteoporotic fracture is low bone mineral density (BMD) [1,2], which is under strong genetic control, with heritability estimates ranging from 50% to 80% [3-5]. Osteoporosis is associated with peak bone mass achieved during early adulthood and the rate of bone loss later in life. Identification of genes underlying peak BMD may be useful in predicting the risk of low bone mass and osteoporosis in later life.

The Wnt/beta-catenin signaling pathway plays an important role in skeletal development and growth [6,7]. In this pathway, Wnt signaling is transmitted to the nucleus when Wnt binds to the frizzled receptor/low-density lipoprotein receptor-related protein (LRP) 5 or 6 coreceptor complex. Variations in LRP5 and frizzled genes are associated with bone mass variation [8-12]. The signaling function of the Wnt/beta-catenin signaling pathway is antagonized by secreted frizzled related proteins (sFRPs). One member of the sFRP family is the frizzled-related protein gene (*FRZB*), which is mapped to human chromosome 2q31-33 and consists of 6 exons and 5 introns [13]. FRZB (also called secreted frizzled-related protein 3, sFRP3), which contains a cysteine-rich domain that is highly homologous to the ligand-binding domain of frizzled receptors [14], antagonizes Wnt signaling by binding to Wnt ligands in the extracellular space and sequestering them from their frizzled receptors [15]. Many studies have reported that sFRP3 might play a role in skeletal morphogenesis [16] and protect against the development of cartilage damage in human disease and in mouse models [17-21]. *Frzb*-knockout mice, although phenotypically normal at birth, experience an accelerated loss of cartilage integrity [18] and have increased cortical bone thickness and density [22]. Loughlin et al. [17] found that Arg324Gly substitution within the *FRZB *gene was associated with hip osteoarthritis, and that a haplotype coding for Arg200Trp and Arg324Gly substitutions in *FRZB *was a strong risk factor for primary hip osteoarthritis in females. However, before now, the relationship between *FRZB *gene polymorphisms and BMD variation has never been published. In this study, we analyzed the association of four tagging single nucleotide polymorphisms (tagSNPs) and the haplotypes defined in the *FRZB *gene with peak BMD variation in the spines and hips of two relatively large samples of Chinese female-offspring nuclear families and male-offspring nuclear families. The two SNPs rs288326 (Arg200Trp) and rs7775 (Arg324Gly) analyzed by Loughlin et al. [17] were not selected in this study because of their low minor allele frequencies (MAFs) of 0 and 0.011, respectively, in the Chinese population.

## Methods

### Subjects

The study was approved by the Ethics Committee of Shanghai Jiao Tong University Affiliated Sixth People's Hospital. All of subjects involved in this study were recruited by the Department of Osteoporosis from the local population of Shanghai City and signed informed consent documents before entering the study. We recruited 401 female-offspring nuclear families, composed of both parents and at least one healthy daughter, totaling of 1,260 individuals, and 427 male-offspring nuclear families, composed of both parents and at least one healthy son, totaling 1,296 individuals. All of the nuclear families have lived in Shanghai for at least three generations and were recruited using public advertising from universities and social and community centers from 2001 to 2007. All of the participants belong to the Chinese Han ethnic group, identified by a combination of self-reporting and identification cards. The average size of the investigated female-offspring nuclear families was 3.14 people, in which 348, 50, 2, and 1 families had one, two, three, and four daughters, respectively [23,24]. In the investigated male-offspring nuclear families, the average family size was 3.03, in which 412 and 15 families had one and two sons, respectively. All of the recruited daughters and sons were 18-44 years old and healthy, and the daughters were premenopausal. For each participant, we also collected information on age, sex, medical history, family history, physical activity, dietary habits, smoking history, and other lifestyle variables. The exclusion criteria for daughters have been detailed elsewhere [23,24]. The following exclusion criteria were used for sons to minimize any known potential confounding effects on the studied phenotypes: (1) serious residuals from cerebral vascular disease; (2) diabetes mellitus; (3) chronic renal disease; (4) serious chronic liver disease or alcoholism; (5) significant chronic lung disease; (6) corticosteroid therapy at pharmacologic levels for >6 months; (7) treatment with anticonvulsant therapy for >6 months; (8) evidence of other metabolic or inherited bone diseases, such as hyper- or hypoparathyroidism, Paget's disease of bone, osteomalacia, osteogenesis imperfecta, or others; (9) rheumatoid arthritis or collagen disease; (10) recent major gastrointestinal disease (within the past year), such as peptic ulcer, malabsorption, chronic ulcerative colitis, regional enteritis, or any significant chronic diarrhea state; (11) significant disease of any endocrine organ that would affect bone mass; (12) hyperthyroidism; and (13) any neurological or musculoskeletal condition that would be a non-genetic cause of low bone mass.

### BMD measurements

BMD (g/cm^2^) of the lumbar spine (L1-L4) and left proximal femur including the total hip and femoral neck, was measured by dual-energy X-ray absorptiometry (DXA) on a Hologic QDR 2000 (Hologic, Bedford, MA, USA) for female-offspring families and on a Lunar Prodigy (GE Lunar Corp., Madison WI, USA) for male-offspring families. Both of the scanners were calibrated daily, and the coefficient of variability (CV) values of the DXA measurements at L1-4, total hip, and femoral neck were 0.9%, 0.8%, and 1.93%, respectively, for the Hologic QDR 2000 [23,24] and 1.39%, 0.7%, and 2.22%, respectively, for the Lunar Prodigy [25]. The long-term reproducibility of the DXA data during the trial, based on weekly repeated phantom measurements, was 0.45%. Weight and height were measured using a calibrated balance beam scale and a calibrated stadiometer, respectively.

### SNP selection and genotyping

SNPs located within the FRZB gene were selected from NCBI's LocusLink http://www.ncbi.nlm.nih.gov/LocusLink/ and HapMap http://hapmap.ncbi.nlm.nih.gov/. Polymorphisms spanning the *FRZB *gene were selected from the SNPs resource based on the estimated pairwise linkage disequilibrium (LD), r^2^, between all common SNPs. The tagging SNPs were selected from each bin such that they constituted a minimal set of highly informative markers while minimizing redundant data. SNPs in *FRZB *gene were evaluated for the following criteria: (1) validation status, especially in Chinese; (2) degree of heterozygosity (MAFs > 10%); (3) r^2 ^= 0.8; and (4) being tagSNPs. Using these criteria, we selected four tagSNPs (rs6433993, rs409238, rs288324, and rs4666865), which are located in intron 1, intron 2, intron 5, and 3' near gene, respectively.

Genomic DNA was extracted using a standard phenolchloroform extraction procedure. DNA concentration was assessed by UV-VIS spectrophotometry (Uvmini-1240). SNPs rs409238, rs288324, and rs4666865 were genotyped using the TaqMan assay, with the primer and probe sequences optimized using the SNP assay-by-design service of Applied Biosystems. One allelic probe was labeled with FAM dye and the other with fluorescent VIC dye. The genotype for every sample was named according to the ratio of the fluorescence intensities of the two dyes. The sequences of the PCR primers for the three SNPs are presented in Table 1. Reactions were performed in a Mx3000P Real-Time PCR System (STRATAGENE, CA) with 20 ng genomic DNA in a 10-μl reaction volume in every well. It was not possible to genotype rs6433993 by TaqMan, so it was genotyped by polymerase chain reaction and restriction fragment length polymorphism (PCR-RFLP). The primers for genotyping this polymorphism are summarized in Table 1. Genomic DNA (0.1-0.3 μg) was amplified in a 10-μl final reaction mixture. After amplification, the PCR product was digested with restriction endonuclease *Eco *47I (*Ava*) and electrophoresed in 2.5% agarose. The presence and absence of the restriction enzyme site were defined as G (60 bp+112 bp) and A (172 bp), respectively.

**Table 1:** Information and the primer and probe sequences for the studied SNPs in the *FRZB *gene

dbSNP	Polymorphism	Domain	primer sequence 5'-3'	TaqMan probe sequence
rs6433993	A/G	intron 1	F: CTGCTGAAATTAACATACCTGACCTGGATTT AAATATATACCAGTTCAGTGTGATAGGTC R: CAAAATTTGTAAATGATAAGCATCCTA	
rs409238	A/G	intron 2	F: TCTGGAGCACCTTTGGAACAG R: GGGAACATTAGTGTAAGTCAGATGCT	VIC-CGCCAAGAACAGGTT FAM-CGCCAAGAGCAGGTT
rs288324	A/G	intron 5	F: CTTGAAATGCATCTCCCTTTTGACA R: GTAAGGAGAAACTACCCTCCAGTAAGT	VIC-TCTTCTCCCCTTTAGTAGAT FAM-TCTCCCCTTCAGTAGAT
rs4666865	A/G	3'near gene	F: TGGTTCTACTAATAGCACACATGTAAATGG R: CTTAGAGCCTGTGCCAATTACTTG	VIC-CAGAGAATGAAACTTT FAM-CAGAGAATGAGACTTT

The rs6433993 was genotyped by polymerase chain reaction and restriction fragment length polymorphism (PCR-RFLP) and did not use a TaqMan probe

### Statistical analysis

Regular statistical analyses were performed using SPSS version 11.0 (SPSS, Chicago, IL, USA). Allele frequencies for each SNP were calculated by allele counting, and the Hardy-Weinberg equilibrium (HWE) was assessed by χ^2 ^analysis. The heritability estimates were conducted using the linear regression of parents' mean value and offspring's value of every phenotype (described at http://www.heritability.com). Statistical power was estimated by the Piface program (version 1.65 http://www.math.uiowa.edu/~rlenth/Power/) in our current sample size according to the MAF of every genotype and the variation in BMD. Linkage disequilibrium block structure was examined using the Haploview verision 3.2 [26] in unrelated parents. Haplotypes were constructed from the population genotype data by the algorithm of Stephens using Phase software version 2.0.2 [27].

The quantitative transmission disequilibrium test (QTDT) program (available at http://www.sph.umich.edu/csg/abecasis/QTDT/), a powerful family-based test for nuclear families of any size, was used to test for population stratification, total association, within-family association, and linkage between each SNP and haplotypes with BMD phenotypes. Total association is sensitive to population stratification, and with-family association is unaffected by population stratification and significant only when linkage disequilibrium is present. Because there was the possibility of generating false-positive results in the present study, to assess the reliability of the results, permutations (1000 simulations) were performed to generate the empirical *p *values [24,28,29]. Linkage tests are based on the identity-by-descent (IBD) relationships of genotypes among family members.

In all of the statistical analyses, raw BMD values were adjusted by age, height, and weight as covariates. Sex was not used as a covariate because male- and female-offspring nuclear families were tested separately, and the parents' phenotypes were excluded in the QTDT. By performing the Shapiro-Wilks test, we found the BMD data in our sample generally did not deviate significantly from the normal distribution. In all analyses, *P *< 0.05 was considered significant.

## Results

### Basic characteristics of the subjects

A total of 1,260 individuals from 401 female-offspring nuclear families, comprising 802 parents and 458 female offspring and 1,296 individuals from 427 male-offspring nuclear families, comprising including 854 parents and 442 male offspring were recruited. From these 427 male offspring nuclear families, samples from 15 individuals could not be amplified in the PCR due to the poor quality of DNA, and 12 sons deviated from the Mendelian inheritance. Therefore, we had 400 male-offspring nuclear families for our analyses. The basic characteristics of the study subjects are presented in Table 2. Parents in female-offspring nuclear families seemed to have lower BMD values than parents in male-offspring nuclear families because of the different DXA devices used in the two cohorts. Because we analyzed the association between *FRZB *genotypes and peak BMD variation in female-offspring nuclear families and male-offspring nuclear families separately, and the effects of parents' phenotypes were excluded in the QTDT, we did not transform the BMD data. Peak BMD is thought to be under strong genetic control. In our male samples, the heritability estimates for peak BMD in the spine, total hip and femoral neck were 0.565, 0.693, and 0.702, respectively, which agreed with the heritability for peak BMD in the female samples [30].

**Table 2:** Basic characterstics of the study subjects

	Female-offspring nuclear families^a^			Male-offspring nuclear families^b^		
	
	Father	Mother	Daughter	Father	Mother	Son
n	401	401	458	400	400	415
Age (years)	62.4 ± 6.6	62.4 ± 6.6	31.4 ± 5.8	61.1 ± 7.1	58.4 ± 6.3	30.4 ± 6.1
Height (cm)	165.7 ± 10.4	154.6 ± 5.6	159.8 ± 5.1	167.7 ± 6.1	155.9 ± 5.4	172.9 ± 5.9
Weight (kg)	68.3 ± 10.6	59.2 ± 8.6	55.0 ± 8.0	69.7 ± 9.5	58.3 ± 8.3	70.7 ± 10.8
Lumbar spine BMD (g/cm^2^)	0.930 ± 0.149	0.813 ± 0.151	0.960 ± 0.102	1.138 ± 0.171	0.994 ± 0.170	1.138 ± 0.137
Total hip BMD (g/cm^2^)	0.875 ± 0.123	0.749 ± 0.134	0.854 ± 0.108	0.966 ± 0.130	0.869 ± 0.149	1.015 ± 0.137
Femoral neck BMD (g/cm^2^)	0.750 ± 0.115	0.677 ± 0.122	0.776 ± 0.108	0.891 ± 0.132	0.797 ± 0.144	0.998 ± 0.143

All data are presented as mean ± SD for the raw phenotype values without adjustment.

^a ^Female-offspring families were measured by DXA on a Hologic QDR 2000.

^b ^Male-offspring families were measured by DXA on a Lunar Prodigy

### Frequencies of alleles and haplotypes

**Figure 1:**
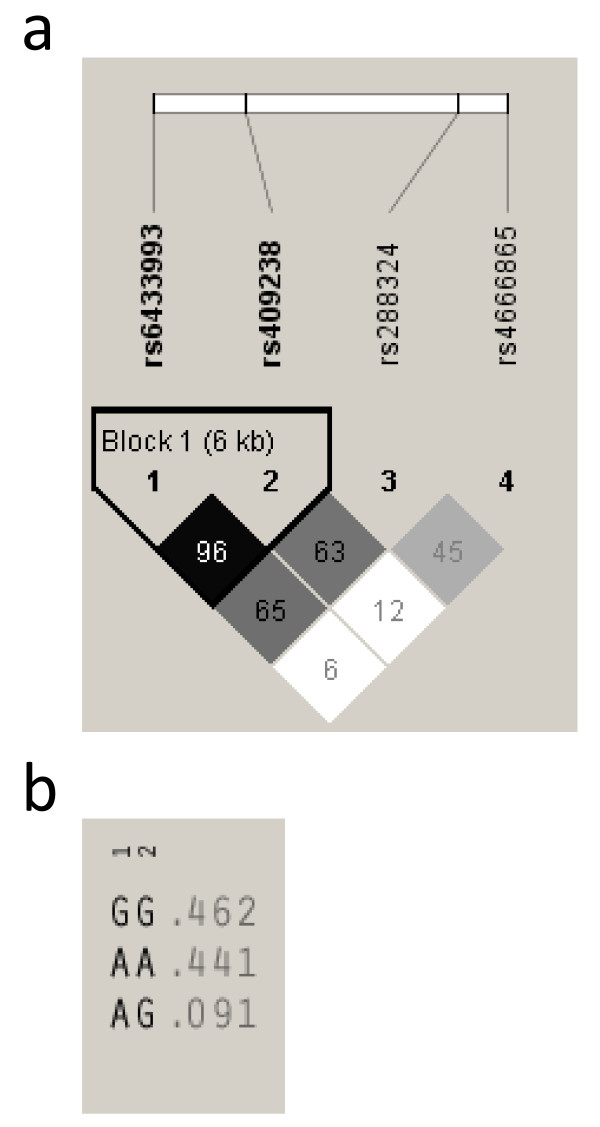
**Linkage disequilibrium (LD) patterns and haplotype frequencies for the *FRZB *gene**. Block structures depicted by Haploview. The increasing degree of darkness of the cells from white to black represents the increasing strength of LD. The values in the cells are the pair-wise degrees of LD indicated by D' × 100 when D' < 1. In the figure, 1 to 4 represent rs6433993 rs409238 rs288324 and rs4666865 respectively. The haplotype frequency is denoted beside each corresponding haplotype and calculated from unrelated parents (n = 1602). **a **LD pattern for the *FRZB *gene. **b **Haplotype frequencies for the *FRZB *gene.

The data of four FRZB SNPs is summarized in Table 1. All of the four SNPs are tagSNPs, although no potential functional missense was found. The SNPs were arranged in the order of rs6433993, rs409238, rs288324, rs4666865 from 5' to 3'. The MAFs were 46.8%, 44.6%, 44.3%, and 16.2% for rs6433993, rs409238, rs288324 and rs4666865, respectively (Table 3). All of the SNPs were in HWE (*P *> 0.05). Based on D' values (*D*' = 0.96), we identified one block with high LD, ranging from intron 1 to intron 2, which included rs6433993 and rs409238 (Figure 1). When the haplotypes were reconstructed by rs6433993 and rs409238, all four possible haplotypes were obtained, with a frequency of 46.2% for the commonest one. Since the least common haplotype had a frequency of only 0.5%, we did not use it for subsequent statistical analysis (Figure 1)

**Table 3:** Frequencies of selected *FRZB *polymorphism in the Chinese population

dbSNP	Genotype	Parents in female-offspring nuclear families (n = 802)	Parents in male-offspring nuclear families (n = 800)	All parents (n = 1602)	MAF (%)
rs6433993	AA	243(0.303)	205(0.256)	448(0.280)	46.8
	AG	405(0.505)	403(0.504)	808(0.504)	
	GG	154(0.192)	192(0.240)	346(0.216)	
rs409238	AA	170(0.212)	133(0.166)	303(0.189)	44.6
	AG	414(0.516)	411(0.514)	825(0.515)	
	GG	218(0.272)	256(0.320)	474(0.296)	
rs288324	AA	169(0.211)	140(0.175)	309(0.193)	44.3
	AG	392(0.489)	409(0.511)	801(0.500)	
	GG	241(0.300)	251(0.314)	492(0.307)	
rs4666865	AA	559(0.697)	558(0.697)	1117(0.697)	16.2
	AG	229(0.286)	224(0.280)	453(0.283)	
	GG	14(0.017)	18(0.023)	32(0.020)	

MAF minor allele frequency

MAFs of each SNP are calculated from all of the parents of the nuclear families

### Association between SNPs and haplotypes with peak BMD

The results of the association of *FRZB *genotypes with peak BMD by QTDT are summarized in Tables 4 and 5. There were 349, 356, 350, and 218 informative female-offspring nuclear families and 303, 315, 313, and 192 male-offspring nuclear families for the TDT analysis at rs6433993, rs409238, rs288324, and rs4666865, respectively. No population stratifications for single SNPs or haplotypes in either female-offspring or male-offspring nuclear families were found. In the total 401 female-offspring nuclear families, no single SNP showed significant evidence of association (including within-family association and total association) with peak BMD in the lumbar spine or hip. As for male-offspring nuclear families, we failed to detect significant within-family association between any single SNP and BMD, although a total association was found between rs4666865 and BMD in the spine (*P *= 0.0299). Furthermore, no significant association was found between haplotypes and BMD in the spine or hip in either female- or male-offspring nuclear families (data not shown). In multiple-parameter tests of 1,000 permutations, the permutations agreed with these within-family association results (*P *> 0.05). In addition, using tests for linkage and tests for linkage while modeling association, no significant results for linkage between each SNP or haplotype and BMD in the spine or hip were observed (data not shown).

**Table 4:** *P *values of QTDT analyses for *FRZB *gene and BMD in female-offspring nuclear families

	rs6433993	rs409238	rs288324	rs4666865
Tests of population stratification				
Lumbar spine BMD	0.2597	0.7082	0.7210	0.6797
Total hip BMD	0.1201	0.4474	0.6278	0.3498
Femoral neck BMD	0.9553	0.5299	0.8853	0.5387
Test of total association				
Lumbar spine BMD	0.5910	0.9239	0.8353	0.3077
Total hip BMD	0.7237	0.6503	0.2945	0.7763
Femoral neck BMD	0.4555	0.2779	0.1819	0.9844
Test of within-family association				
Lumbar spine BMD	0.5047	0.7121	0.8470	0.8338
Total hip BMD	0.1345	0.3765	0.3300	0.5350
Femoral neck BMD	0.6584	0.9779	0.5681	0.6117
*p *1000 permutations of within-family association				
Lumbar spine BMD	0.3950	0.6670	0.7830	0.8090
Total hip BMD	0.1110	0.3520	0.2680	0.5170
Femoral neck BMD	0.6440	0.9780	0.5990	0.6650

BMD values are adjusted for age, height, and weight

**Table 5:** *P *values of QTDT analyses for *FRZB *gene and BMD in male-offspring nuclear families

	rs6433993	rs409238	rs288324	rs4666865
Tests of population stratification				
Lumbar spine BMD	0.5236	0.7656	0.6731	0.8238
Total hip BMD	0.7361	0.6912	0.9392	0.8309
Femoral neck BMD	0.8698	0.7332	0.8995	1.0000
Test of total association				
Lumbar spine BMD	0.5978	0.2937	0.2672	**0.0299**
Total hip BMD	0.5233	0.6343	0.7110	0.1164
Femoral neck BMD	0.8093	0.8872	0.7740	0.2963
Test of within-family association				
Lumbar spine BMD	0.4155	0.4277	0.3610	0.3712
Total hip BMD	0.9793	0.9271	0.8014	0.5332
Femoral neck BMD	0.9752	0.7143	0.8034	0.6109
*p *1000 permutations of within-family association				
Lumbar spine BMD	0.3860	0.3700	0.3680	0.2780
Total hip BMD	0.9740	0.7350	0.8280	0.6520
Femoral neck BMD	0.9800	0.9290	0.8310	0.5290

BMD values are adjusted for age, height and weight. Bold indicates significant *p *values (*p *< 0.05)

## Discussion

To the best of our knowledge, this is the first study to test the possible influence of the *FRZB *gene on peak BMD variation in the Chinese population. After adjusting for the covariates of age, height, and weight, we failed to find a significant within-family association of any SNP or haplotype with peak BMD variation at the spine or the hip in females or males.

In this study, we performed QTDT to test the relationship between *FRZB *SNPs and haplotypes and BMD. Although QTDT has a lower power to detect allelic association than the population-based association approach, it is robust to population stratification, which avoids the false-positive or false-negative results that may easily occur in association analyses because of an admixed and stratified population. In our previous studies, the number of female-offspring nuclear families offered more than 80% power to test a candidate gene as a quantitative trait locus (QTL), explaining about 10% of BMD variation [23,24,30]. Furthermore, previously our method was successful in identifying genetic variation associated to BMD [23,24]. Based on the power calculation, the present study had sufficient power to detect any association between a SNP and peak BMD in males and females. Moreover, the MAF of three of the four SNPs was > 44%, and the heterozygosity was high in our population, except for rs4666865, which may be explained by the lower MAF of 16.2%. With greater heterozygosity, more information can be derived from families in the QTDT analysis. Although males and females may share a genetic contribution to BMD, many candidate genes have been observed to have different effects on BMD in males and females [31-33]. In this study, we found that heritability for peak BMD was similar in males and in females; however, we separately analyzed the association between *FRZB *genotypes and peak BMD in males and females, which might avoid the impact of gender mixture on results. Because of the advantages mentioned above, the possibility of false-negative findings in our study was minimized.

Over the past few years, the canonical Wnt pathway has been shown to play a substantial role in the regulation of skeletal development [34] and to affect osteoblast differentiation [35]. In this pathway, Wnt interacts with the frizzled/LRP5 or 6 coreceptor complex, which leads to the stabilization of beta-catenin and its accumulation in the nucleus, where it coactivates TCF/Lef transcription factors [14]. A loss or gain of function in the Wnt coreceptor LRP5 is associated with osteoporosis or high bone mass, respectively [36-39]. One family member of the frizzled gene family, frizzled homolog 1, was also found to contribute to the genetic regulation of bone mass and geometry [12]. *FRZB *inhibits Wnt signaling by either binding to Wnts and preventing them from activating frizzled receptors or directly inactivating the receptors [15]. Loughlin et al. [17] found functional variants within *FRZB *gene were associated with hip osteoarthritis in females, possibly through a reduced ability in antagonizing Wnt signaling. Since osteoarthritis and osteoporosis are shown to be inversely related [40] and increased Wnt signaling is associated with high bone mass [36-38], loss of function in *FZRB *may lead to higher bone density. However, in this study, we did not find an association between *FRZB *genotypes and peak BMD in females or in males. These results demonstrate that *FRZB *may not be required for the attainment of peak BMD and suggest that other members of the sFRP family may compensate for reduced *FRZB *activity [22].

Hsu et al. [41] performed a genome-wide scan for total hip BMD from a large cohort in China that revealed a significant QTL on chromosome 2q24.3 for total BMD (LOD = 3.65). *FRZB *is a potential candidate gene in the chromosome 2q QTL region. However, in our study, we did not observe any association between *FRZB *polymorphisms and peak BMD in the spine or hip. Other genes in this region do contribute to the BMD variation, as our previous study has identified [24]. In addition, we focused on the possible influence of the *FRZB *genotypes on peak BMD variation; whether the gene plays a more important role in affecting bone loss requires further study.

Our study had several limitations. First, because we selected the most informative SNPs with high MAF and r^2^, some causal variants might be missed. We also did not select the SNPs rs288326 (Arg200Trp) and rs7775 (Arg324Gly), which have been analyzed in other studies [17,42,43], due to their MAFs of only 0 and 0.011, respectively, and hence their poor informativeness in the Chinese population. Second, although we had two relatively large samples of nuclear families, the number of families that had more than one child was small, making the power to detect linkage between SNPs and BMD modest.

## Conclusions

In summary, we tested the association and linkage between SNPs and haplotypes in the *FRZB *gene with peak BMD variation in the spines and hips of individuals in Chinese nuclear families. Our results failed to support the hypothesis that *FRZB *is a QTL associated with peak BMD variation in the spines and hips of either the female or male Chinese populations. Because ethnic difference in *FRZB *genotypes may exist, other studies in different population are required to confirm these results.

## Competing interests

The authors declare that they have no competing interests.

## Authors' contributions

GG designed the study, carried out all statistical analyses and drafted the manuscript. ZLZ conceived and designed the study and revised the manuscript. JWH guided the work of the genetics laboratory and guaranted and confirmed the quality of the genetic data. HZ and HY guided the work of the genetics laboratory. WWH, JMG, WZF, YQH, ML, and YJL carried out the field work including sample and data collection. JBY coordinated the DNA sample collection. All authors read and approved the final manuscript.

## Pre-publication history

The pre-publication history for this paper can be accessed here:

http://www.biomedcentral.com/1471-2350/11/1/prepub
